# Bradyarrhythmias Associated with Oncologic Treatment—An Updated Review

**DOI:** 10.3390/cancers18101556

**Published:** 2026-05-11

**Authors:** Jakub Stępień, Julita Stępniak-Bielecka, Anna Ciołek, Jędrzej Piotrowski, Aleksandra Kryca, Grzegorz Piotrowski

**Affiliations:** 1 Department of Cardiooncology, Medical University of Lodz, 90-647 Lodz, Poland; julita.stepniak@kopernik.lodz.pl (J.S.-B.); anna.szczepanska@stud.umed.lodz.pl (A.C.); jedrzej.piotrowski@kopernik.lodz.pl (J.P.); aleksandra.kryca@kopernik.lodz.pl (A.K.); grzegorz.piotrowski@umed.lodz.pl (G.P.); 2 Department of Cardiology, Nicolaus Copernicus Memorial Hospital, Pabianicka 62, 93-513 Lodz, Poland

**Keywords:** bradyarrhythmias, conduction disturbances, atrioventricular block, sinus node dysfunction, cardio-oncology, cardiotoxicity, immune checkpoint inhibitors, ALK inhibitors, VEGFR-TKIs, proteasome inhibitors

## Abstract

**Background/Objectives**: Bradyarrhythmias are an under-recognized component of cancer therapy-related cardiovascular toxicity. As survivorship improves and exposure to targeted and immune therapies increases, clinicians increasingly face clinically relevant sinus node dysfunction and atrioventricular (AV) conduction disease that may interrupt otherwise effective oncologic treatment. We aimed to synthesize evidence on the incidence, phenotype, mechanisms, and management of bradyarrhythmias across major anticancer drug classes. **Methods**: We performed a narrative review of PubMed and major clinical guidelines, prioritizing prospective trials and large observational cohorts for incidence estimates, and case reports/series for phenotype and management details. Regulatory prescribing information and pharmacovigilance datasets were consulted to complement trial data. Evidence was organized by drug class and prototypical agents. **Results**: Bradyarrhythmias ranged from transient sinus bradycardia to high-grade atrioventricular block requiring pacing. The most severe phenotype was associated with immune checkpoint inhibitor-related myocarditis, whereas ALK inhibitors, thalidomide, antimetabolites—particularly 5-fluorouracil—and taxanes showed more reproducible signals for sinus bradycardia. Bradyarrhythmic events were also described with proteasome inhibitors, BTK (Bruton tyrosine kinase) inhibitors, anthracyclines, platinum compounds, high-dose cyclophosphamide, corticosteroids, and other agents, but the strength of evidence varied from regulatory or cohort-based data to isolated case reports. **Conclusions**: Bradyarrhythmias during cancer therapy are heterogeneous but clinically consequential. Recognition of class-specific patterns, proactive ECG/electrolyte monitoring, and context-specific management (e.g., drug interruption/dose modification, pacing when indicated, risk-factor control) can minimize therapy interruption while maintaining oncologic efficacy.

## Simple Summary

Over the past few decades, cancer treatment has improved greatly, helping many patients live longer. However, because patients now receive cancer treatments for longer periods of time, they may also be exposed to unwanted effects on the heart for a longer period. One possible heart-related side effect is a slow heart rate, which may cause symptoms, require closer monitoring, or sometimes lead to changes in cancer treatment. In this review, we describe cancer medicines that have most often been linked to a slow heart rate and discuss how this problem may affect patient care.

## 1. Introduction

Over the past few decades, advances in oncologic therapies have markedly improved the prognosis and survival of patients with cancer. The growing population of cancer patients, however, faces an unintended consequence: increased exposure to the cardiotoxic effects of anticancer drugs and therapies. Modern-day cardio-oncology focuses almost entirely on predicting and reducing cardiotoxicity, as it may not only worsen quality of life but also compromise the continuation of otherwise effective oncologic treatment.

Within the broad spectrum of cardiotoxicities, the most attention has been devoted to heart failure and tachyarrhythmias. In contrast, bradyarrhythmias remain an underrecognized but clinically significant topic. These disturbances of cardiac conduction and sinus node dysfunction may, even when properly treated, complicate or even lead to discontinuation of effective anticancer treatment. The severity of these disturbances is graded according to the NCI CTCAE, as shown in Table 1.

This review aims to outline the potential bradyarrhythmic adverse effects associated with different classes of anticancer drugs, summarize the current evidence, and discuss their clinical implications for cardio-oncology practice.

## 2. Materials and Methods

This narrative review was based primarily on PubMed/MEDLINE searches, complemented by major cardio-oncology and pacing guidelines, regulatory prescribing information from the U.S. Food and Drug Administration and European Medicines Agency, and selected pharmacovigilance analyses. PubMed/MEDLINE was searched from January 1980 to November 2025. The initial search combined terms related to bradyarrhythmias and conduction disease with terms related to cancer therapy, including: “bradycardia”, “bradyarrhythmia”, “sinus node dysfunction”, “sinoatrial block”, “sinus pause”, “atrioventricular block”, “complete heart block”, “cancer”, “oncology”, “chemotherapy”, “anticancer therapy”, “immunotherapy”, “targeted therapy”, and “cardio-oncology”.

Additional targeted searches were performed for individual drug classes and representative agents when the initial search suggested sparse or heterogeneous evidence. These searches combined the drug or drug-class name with the above bradyarrhythmic terms, including immune checkpoint inhibitors, ALK inhibitors (anaplastic lymphoma kinase), proteasome inhibitors, immunomodulatory imide drugs (IMiDs), VEGFR-TKIs (vascular endothelial growth factor receptor tyrosine kinase inhibitors), RAF/MEK (rapidly accelerated fibrosarcoma/mitogen-activated extracellular signal-regulated kinase) inhibitors, HER2-targeted therapies, BTK inhibitors, anthracyclines, antimetabolites, BCR-ABL TKIs (breakpoint cluster region–Abelson/BCR-ABL1 oncoprotein), endocrine therapies, CDK4/6 (cyclin-dependent kinases 4 and 6) inhibitors, EGFR (epidermal growth factor receptor) inhibitors, plant alkaloids, platinum compounds, alkylating agents, mTOR (mechanistic target of rapamycin) inhibitors, corticosteroids, radiotherapy, and combination regimens. Reference lists of relevant articles were also screened to identify additional reports.

We included English-language original studies, clinical trials, observational cohorts, pharmacovigilance analyses, case series, case reports, major clinical guidelines, and regulatory prescribing information that described bradycardia, sinus node dysfunction, atrioventricular block, or other clinically relevant conduction disturbances during or after anticancer treatment. Prospective trials, larger cohorts, regulatory labels, and pharmacovigilance datasets were prioritized for frequency estimates, whereas case reports and case series were included when they provided clinically relevant information on phenotype, mechanism, recurrence, or management.

Publications were excluded if they did not address anticancer treatment, did not report bradyarrhythmias or conduction disturbances, described only tachyarrhythmias or QT prolongation without bradyarrhythmic manifestations, were not available in English, or provided insufficient clinical detail to support attribution to a specific therapy. Because this was a narrative review rather than a systematic review, no formal meta-analysis or risk-of-bias assessment was performed.

## 3. Results

**Figure 1:**
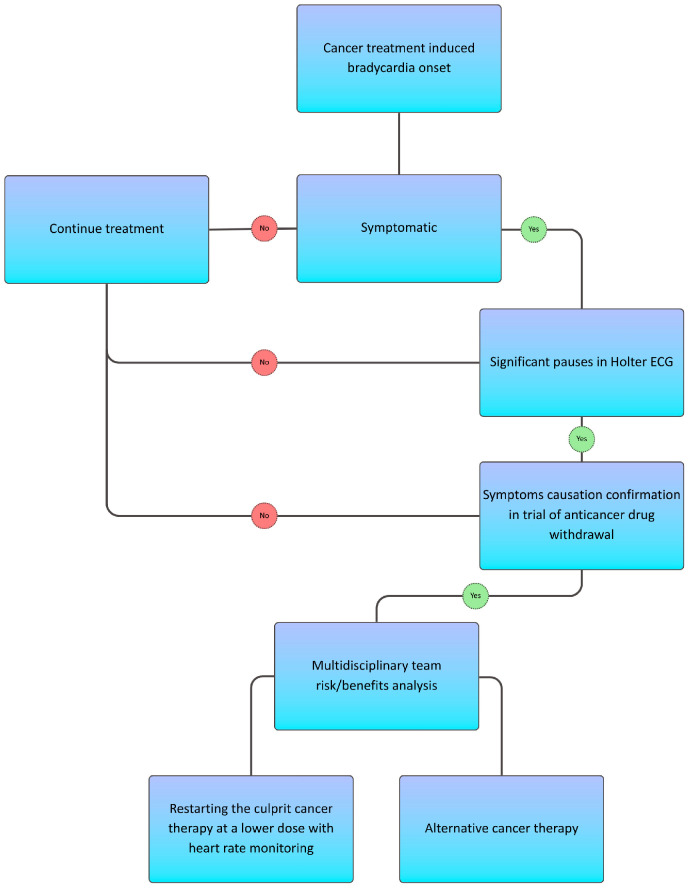
2022 ESC cardio-oncology guidelines for the general management of bradyarrhythmias.

Bradyarrhythmias do occur and may prove clinically significant, with quantifiable incidence available only for select agents and most signals arising from observational cohorts or case reports/series. To aid interpretation, results are organized by drug class and summarized in Table 2. For each agent, we summarize: incidence where numerators/denominators were reported, clinical phenotype (sinus node dysfunction vs. atrioventricular block), mechanistic context, and management. If no specific management is described, treatment should follow standard cardio-oncology guidelines, as shown in Figure 1. 

### 3.1. Immune Checkpoint Inhibitors (ICI)

Immune checkpoint inhibitors (ICIS) target inhibitory receptors such as PD-1 (programmed cell death protein 1) and its ligand PD-L1 (programmed death-ligand 1), as well as CTLA-4 (cytotoxic t-lymphocyte-associated protein 4). Their use has transformed outcomes across multiple solid and hematologic malignancies, but they can provoke immune-related adverse events that involve the cardiovascular system. A representative PD-1 inhibitor widely used across tumor types is pembrolizumab, a human IGG4 monoclonal antibody directed against the PD-1 receptor [1]. Its antineoplastic effect stems from blockade of PD-1–ligand interactions, thereby restoring antitumor T-cell activity [2]. Owing to this versatile mechanism, pembrolizumab has broad oncologic indications, including non-small-cell lung cancer [3], melanoma [4], head and neck squamous-cell carcinoma [5,6], renal-cell carcinoma [7], and urothelial carcinoma [8]. The most clinically serious cardiotoxicity associated with PD-1/PD-L1 inhibitors—including pembrolizumab—is myocarditis, which, although infrequent, is often fulminant and carries substantial mortality [9,10,11]. In the setting of myocarditis, conduction disturbances—including third-degree atrioventricular (AV) block—are not uncommon. Complete AV block is encountered more often than sinus node dysfunction, consistent with the preferential inflammatory involvement of the AV node and His-Purkinje system rather than the sinoatrial node [12]. In a single-center cohort of patients with ICI-associated myocarditis, complete heart block occurred in seven of 34 cases (20.6%) and was associated with worse outcomes [13]. Case-based reviews of published reports have described complete heart block in approximately 25–28% of reported ICI-myocarditis cases, although these estimates are vulnerable to publication and reporting bias [14,15]. Management follows cardio-oncology and pacing guidance: hold ICI, initiate high-dose corticosteroids to treat myocarditis, provide temporary pacing when needed, and consider permanent pacemaker implantation if high-grade block persists despite resolution of the acute inflammatory process [9,12].

### 3.2. Anaplastic Lymphoma Kinase Inhibitors (ALK-TKI)

Across ALK-TKIs, bradycardia is a common occurrence, often occurring early after initiation and typically reversing with dose interruption or reduction; a permanent pacemaker is not part of routine management and should be considered only in rare cases of persistent or clinically significant high-grade atrioventricular block after reversible causes have been excluded [16]. Crizotinib is a tyrosine kinase inhibitor whose mechanism of action involves blocking the activity of ALK (anaplastic lymphoma kinase), ROS1 (ROS proto-oncogene 1 receptor tyrosine kinase), and MET/HGFR (Mesenchymal–Epithelial Transition protooncogene/hepatocyte growth factor receptor). It is used in the treatment of tumors with activation of these pathways, primarily non-small cell lung cancer (NSCLC) [17], anaplastic large cell lymphoma (ALCL) [18,19], and the rare [20] inflammatory myofibroblastic tumor (IMT) [21,22].

The reported incidence of bradycardia during crizotinib therapy varies between studies, from 13% [23] to 41.9% when counting patients with at least one episode of HR < 60 bpm [16].

In one study addressing the issue of bradycardia associated with crizotinib, a statistically significant increase in the number of treatment responders was observed in the group that developed bradycardia [24]. An animal study has shown that crizotinib inhibits the funny current (I_f_), which is essential for the pacemaking function of the sinoatrial node, thereby offering a plausible explanation for the occurrence of bradycardia [25]. Some authors hypothesize that bradycardia may be a class effect of tyrosine kinase inhibitors [16].

**Figure 2:**
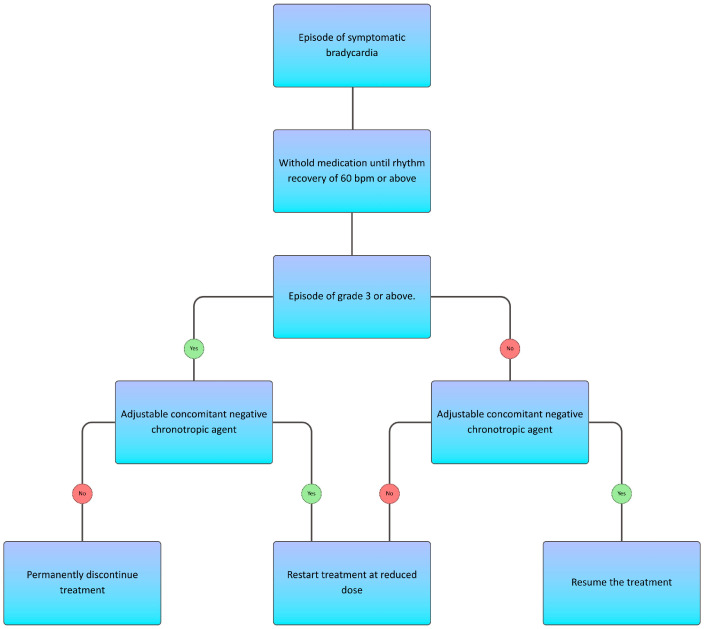
Management algorithm for bradycardia during crizotinib therapy, adapted from the U.S. FDA ((XALKORI^®^) crizotinib; Pfizer Inc., New York, NY, USA) prescribing information.

Owing to the frequency of this adverse event, specific management algorithms have been established for crizotinib-induced bradycardia. In patients without concomitant medications that could explain the reduction in heart rate, dose reduction is recommended for symptomatic bradycardia. Discontinuation of crizotinib is indicated in life-threatening cases. If a concomitant drug potentially responsible for, or contributing to, bradycardia is identified, it should be discontinued. Under these circumstances, crizotinib may be resumed at the prior dose once symptomatic bradycardia resolves, or at a reduced dose following a life-threatening episode [23]. The dose-reduction algorithm is presented graphically in Figure 2.

Alectinib is a selective anaplastic lymphoma kinase (ALK) inhibitor [26]. It is most frequently used for the treatment of ALK-positive non-small cell lung cancer [27]. The most common cardiotoxic complication of alectinib treatment is bradycardia, which is typically reversible and not associated with the impairment of left ventricular ejection fraction [28]. In a prospective study of 47 patients, the incidence of bradycardia was reported as 42% [28]. The FDA (U.S. Food and Drug Administration) label reports bradycardia as an adverse event in 11% of patients; however, in up to 20% of patients with available serial ECGs, post-dose heart rates of less than 50 beats per minute were observed [29]. The mechanism of alectinib-induced bradycardia is not yet entirely clear; however, a dose-dependent effect has been described [28]. Management guidelines for alectinib-associated bradycardia are similar to, but stricter than, those for crizotinib, and include permanent discontinuation of the drug if no concomitant bradycardia-inducing medication is identified or if the event is life-threatening. Resumption of treatment at a reduced dose is possible only if a concomitant bradycardia-inducing drug has been identified and discontinued [29].

Ceritinib is a selective inhibitor of anaplastic lymphoma kinase, demonstrating efficacy in the treatment of ALK-positive non-small cell lung cancer [30]. In a study involving 255 patients receiving ceritinib, cardiotoxicity was primarily manifested by QT interval prolongation (4%), followed by bradycardia, which occurred in 3% of patients [31]. In cases of symptomatic bradycardia, treatment should be withheld, concomitant medications contributing to the event should be corrected, and therapy may be resumed at a reduced dose. In the event of life-threatening bradycardia without contributing concomitant medications, permanent discontinuation of ceritinib is recommended [32].

### 3.3. Proteasome Inhibitors

Proteasome inhibitors—bortezomib, carfilzomib, and ixazomib—are integral to modern multiple myeloma regimens, which has prompted closer scrutiny of their cardiovascular safety in clinical trials and real-world practice. Bradyarrhythmias appear uncommon but documented across the class—most notably with bortezomib. Bortezomib is a reversible 26s proteasome inhibitor [33]. It is used primarily in multiple myeloma [34] and mantle-cell lymphoma [35]. In observational and pooled clinical-trial data, the most common cardiovascular adverse event is hypertension, followed by heart failure and then arrhythmia (without specific breakdown for bradyarrhythmias) [36,37]. The incidence of bradyarrhythmias is not well-defined, but case reports describe complete atrioventricular block [38,39,40] and sinus node dysfunction [41]. The pathogenesis may involve autonomic neuropathy, although the exact mechanism remains uncertain [42].

### 3.4. Immunomodulatory Imide Drugs

Immunomodulatory imide drugs, as a group, consist of the founding agent, thalidomide, and its newer analogs: lenalidomide and pomalidomide. First thalidomide, and later its analogs, were found to be potent anticancer agents, nowadays usually used in the treatment of multiple myeloma [43]. Its mechanism of action is heterogeneous and includes the inhibition of the Tumor Necrosis Factor, upregulation of T and NK (natural killer) lymphocytes, and modulation of interleukin production [44]. This kind of activity in multiple myeloma is achieved through binding with Cereblon [45,46]—a signal protein that “marks” damaged proteins for degradation by proteasomes [44].

In a retrospective study of 96 patients with multiple myeloma receiving thalidomide, heart rate < 60/min occurred in 53% of patients, and symptom-related bradycardia developed in 19% [47]. Regarding atrioventricular block, only case reports are described in the literature [48,49].

Some authors suggest that the overactivity of the parasympathetic system, due to TNF-alpha (tumor necrosis factor alpha) downregulation, is a cause of thalidomide-induced sinus bradycardia [47]. Still, the hypothesis has yet to be proven.

For the newer analogs, lenalidomide and pomalidomide, bradyarrhythmias appear rare, with only isolated cases of sinus bradycardia reported in small cohorts [50,51].

The bradyarrhythmic effect of the group has been recognized in recent cardio-oncology guidelines; however, no specific treatment has been formulated [9].

### 3.5. VEGF Inhibitors (VEGFR-TKI Vascular Endothelial Growth Factor Receptor Tyrosine Kinase Inhibitor)

VEGF-pathway tyrosine-kinase inhibitors are a widely used class of anti-angiogenic agents in solid tumors, whose cardiovascular profile extends beyond hypertension and QT effects to occasional rhythm disturbances. We focus on pazopanib because its bradycardia signal is best documented with clear vital-sign vs. adverse-event reporting. Pazopanib is an oral, multitarget tyrosine kinase inhibitor with substantial anti-angiogenic activity [52,53]. It is used primarily as an anticancer agent in renal-cell carcinoma [54] and soft-tissue sarcoma [55]. Pazopanib-associated cardiovascular adverse effects include hypertension, followed distantly by heart failure and QT prolongation [55,56,57]. Bradyarrhythmias have also been observed. According to the U.S. FDA prescribing information, vital-sign bradycardia (heart rate < 60 bpm) occurred in 19% of pazopanib-treated patients in both the randomized RCC (renal-cell carcinoma) and STS (soft-tissue sarcoma) trials, whereas bradycardia reported as an adverse reaction occurred in ~2% of patients [58]. No drug-specific algorithm has been established for bradycardia; management follows general cardio-oncology guidelines.

### 3.6. RAF/MEK Inhibitors

A combination of rapidly accelerated fibrosarcoma (RAF) and mitogen-activated extracellular signal-regulated kinase (MEK) inhibitor treatment has found clinical use in the treatment of melanoma with the BRAF (B-Raf proto-oncogene serine/threonine kinase) V600 mutation [59]. Cardiotoxicity signals of this bigroup usually include hypertension, left ventricular dysfunction and an increased risk of pulmonary embolism [9]. The bradyarrhythmic effect of both groups is poorly defined, and only a few cases have been reported, most of which involve trametinib [60,61]. In a placebo-controlled phase I cardiac electrophysiology study in patients with solid tumors treated with trametinib, regulatory ECG analyses indicate modest Holter-derived effects on heart rate and atrioventricular conduction, suggesting that trametinib may exert a measurable but usually subclinical influence on cardiac rate control and PR interval [62]. The mechanism underlying this effect remains unclear. The rat sarcoma viral oncogene homolog (RAS)–rapidly accelerated fibrosarcoma (RAF)–mitogen-activated extracellular signal-regulated kinase (MEK)–extracellular signal-regulated kinase (ERK) pathway activation may promote fibroblast proliferation, myofibroblast differentiation, and collagen deposition [63], while excessive fibrosis can interfere with electrical impulse propagation and promote arrhythmias; however, this should be interpreted as a mechanistically plausible background rather than direct evidence for trametinib-induced sinoatrial or atrioventricular nodal dysfunction. According to the European Medicines Agency summary of product characteristics for trametinib, bradycardia is classified as common (≥1% to <10%) [64]. Management of bradycardia after RAF/MEK inhibitors follows standard protocol.

### 3.7. HER2-Targeted Therapies

Within HER2-targeted therapies, trastuzumab is the prototypical agent with the largest cardio-oncology evidence base. Trastuzumab is a recombinant, humanized igg1 monoclonal antibody directed against the HER2 (human epidermal growth factor receptor 2) receptor [65]. It is used to treat tumors with overexpression of the target receptor, most commonly breast cancer [65] and gastric/gastroesophageal junction cancer [66]. The most frequent manifestation of trastuzumab cardiotoxicity is heart failure [67,68]. Accordingly, ESC (European Society of Cardiology) guidelines recommend echocardiographic monitoring every three months during therapy [9]. Bradyarrhythmias are rare but not nonexistent, with a few cases describing sinus bradycardia [69]. Their low incidence has not prompted the development of drug-specific management algorithms.

### 3.8. Bruton Tyrosine Kinase Inhibitors (BTK-I)

This section focuses on ibrutinib, the first-in-class agent, because most published data on sinus node dysfunction and atrioventricular block come from its use. Ibrutinib is an oral, irreversible first-generation inhibitor of Bruton’s tyrosine kinase, a kinase that is an important mediator in the pathogenesis of B-cell malignancies [70]. The introduction of this drug opened new therapeutic possibilities in cancers such as mantle cell lymphoma [70], chronic lymphocytic leukemia [71], and Waldenström’s macroglobulinemia [72]. Cardiotoxicity associated with ibrutinib most commonly manifests as atrial fibrillation [73,74]. Bradyarrhythmias appear relatively rare, and available data come from pharmacovigilance analyses and case reports. In an analysis of adverse event reports submitted to the FDA FAERS (FDA Adverse Event Reporting System) database, sinus node dysfunction was identified as a potentially important manifestation of ibrutinib-related cardiotoxicity; however, there are currently no studies that quantify the incidence of this event [75]. The literature includes clinical cases describing both severe sinus node dysfunction [76,77] and atrioventricular block during ibrutinib therapy; in several cases, permanent pacemaker implantation was required [78]. In human pluripotent stem cell-derived atrial cardiomyocytes, ibrutinib exhibits direct, atrial-specific toxicity [79]. While this effect has been associated with a higher frequency of atrial fibrillation onset in patients [79], its relationship to sinus node dysfunction and atrioventricular conduction disorders has not been studied. To date, because of the small scale of the phenomenon, no drug-specific algorithms for the management of post-ibrutinib bradycardia or for decisions on rechallenge versus discontinuation have been established. Importantly, after rhythm stabilization and pacemaker implantation, ibrutinib has been successfully reintroduced in a subset of patients, suggesting that appropriate management of bradyarrhythmias can allow effective oncologic therapy to continue [78]. No validated criteria currently guide the decision to resume or discontinue ibrutinib after high-grade conduction disease. However, if bradyarrhythmia is the only clinically relevant reason for treatment interruption, successful permanent pacemaker implantation with adequate device function and good clinical tolerance of pacing may be sufficient to support ibrutinib rechallenge in selected patients, particularly when continued BTK inhibition remains oncologically important. Conversely, switching to an alternative therapy should be considered when conduction disease is accompanied by other clinically significant toxicities that are not addressed by pacing, such as recurrent or poorly tolerated atrial fibrillation, uncontrolled cardiovascular complications, or when an effective safer oncologic alternative is available.

### 3.9. Anthracyclines

Anthracyclines remain foundational across solid and hematologic malignancies, with cardiac risk traditionally framed around cumulative dose-related cardiomyopathy. Yet conduction disturbances and bradyarrhythmias do occur—often early and typically underreported. Below, we take a brief look at their frequency, phenotype, and clinical implications specifically with doxorubicin. Doxorubicin is an anthracycline antibiotic with cytotoxic activity. Doxorubicin acts via DNA intercalation with topoisomerase II poisoning and by promoting reactive oxygen species formation [80,81]. It is most used in the treatment of breast cancer, ovarian cancer, acute leukemias, lymphomas, and soft-tissue sarcomas [82]. The cardiotoxicity of doxorubicin is extensively described in the literature and represents the main factor limiting the use of this drug [82]. It usually manifests as cardiomyopathy; however, arrhythmias are also a not-infrequent presentation [83]. In the only prospective Holter-based series reporting a discrete incidence of bradycardia during doxorubicin therapy, sinus bradycardia occurred in 3.4% of patients, a figure that subsequent reviews have adopted [84]. Cases of complete atrioventricular block were also described [85,86], some transient [87]. No dedicated studies have specifically investigated the mechanisms underlying anthracycline-related bradyarrhythmias [86].

### 3.10. Antimetabolites

Antimetabolites are widely used components of cancer therapy, and their cardiac safety profile is relatively well-characterized; bradyarrhythmias are uncommon but documented. In this review, we focus on 5-fluorouracil and cytarabine as representative agents for discussing bradyarrhythmias within this class.

5-fluorouracil is a fluoropyrimidine antimetabolite that exerts its cytotoxic effect primarily by inhibiting thymidylate synthase and is used mainly to treat colorectal, breast, and head-and-neck cancers [88]. The cardiotoxicity of this drug most commonly manifests as ischemic cardiac events, occurring with a frequency of up to 10%, depending on the dose [9].

Transient sinus bradycardia appears to be relatively common, with a reported frequency of approximately 2–12% in medium-sized studies [89,90,91]. In all of these reports, symptoms occurred during treatment consisting of 5-fluorouracil combined with platinum compounds. The mechanism underlying this adverse effect remains debated and may involve coronary vasospasm—a frequent manifestation of 5-FU (5-fluorouracil) cardiotoxicity [92]—or hypervagotonia [93].

Another agent in this class is cytarabine, a cytosine antimetabolite. It is primarily used in hematologic oncology [67]. Its cardiotoxic effect is not widely described in the literature and is usually limited to case reports concerning drug-induced pericardial diseases [94,95,96], the incidence of which is unknown but generally considered low [9].

To date, the incidence of cytarabine-associated bradycardia has been quantified in only one study involving a moderately sized cohort (*n* = 141), where it occurred in 2.8% of patients [97]. Given the paucity of data, there are currently no definitive conclusions regarding the underlying mechanism, specific prevention, or management should it occur (standard protocol should be followed).

### 3.11. BCR-ABL TKI

BCR-ABL tyrosine-kinase inhibitors (TKIs) are small-molecule, ATP (adenosine triphosphate)-competitive inhibitors of the BCR-ABL1 oncoprotein that transformed outcomes in CML (chronic myeloid leukemia) and are also used in Ph-positive ALL [98,99].

Among BCR-ABL TKIs, imatinib generally exhibits the most favorable cardiovascular profile, dasatinib is notable for pleural effusions and pulmonary arterial hypertension, and nilotinib for QTc prolongation and vascular events [100]. Ponatinib, a third-generation, highly potent BCR-ABL inhibitor developed for resistant disease, has the highest overall cardiovascular risk profile, with arterial occlusive events as the hallmark and most consequential cardiotoxicity (31% 5-year cumulative risk), whereas the most frequent cardiovascular adverse events were hypertension and cardiac arrhythmias [101,102,103]. Hypertension is a common adverse effect across tyrosine-kinase inhibitors; the risk in BCR-ABL TKI is the highest with ponatinib and intermediate with nilotinib, whereas signals are less consistent for imatinib and dasatinib [9,104].

In the PACE safety dataset (*n* = 449), symptomatic bradyarrhythmias requiring pacemaker implantation occurred in 1% of ponatinib-treated patients [105,106].

For the ATP-competitive BCR-ABL TKIs outside ponatinib, bradyarrhythmias are uncommon and largely case-based: with dasatinib, isolated third-degree AV block requiring permanent pacing has been reported [107], with nilotinib, bradycardia appears among serious adverse events in a switch study (1/18 patients) [108].

Bradyarrhythmias observed with ATP-competitive BCR-ABL tyrosine-kinase inhibitors likely mirror the class mechanism described for other TKIs—namely, interference with cardiac ion channels, with additional ischemic substrate from arterial occlusive toxicity—most evident with ponatinib [109,110].

For management, significant bradyarrhythmia on ponatinib should prompt drug interruption and clinical monitoring with dose reduction or discontinuation per label [105].

### 3.12. Endocrine Therapies

Androgen deprivation therapy (ADT) is used as an adjuvant or neoadjuvant therapy for prostate cancer. Agents used for ADT include GnRH agonists or antagonists; the latter have a safer cardiovascular safety profile [9]. The most important manifestations of cardiotoxicity in patients receiving ADT are hypertension, diabetes, ischemic heart disease, and cardiac dysfunction. QT interval monitoring during therapy is advised if an abnormal QT interval has been observed before treatment [9]. Triptorelin is a gonadotropin-releasing hormone (GnRH) agonist that initially stimulates and then down-regulates pituitary GnRH receptors, leading to the suppression of LH/FSH (luteinizing hormone/follicle-stimulating hormone), and is mainly used for the treatment of prostate cancer [111]. In pharmacovigilance analyses of prostate-cancer therapies based on FAERS, triptorelin was associated with markedly increased reporting odds for complete atrioventricular block [112]. Relugolix is an orally active, non-peptide GnRH-receptor antagonist that blocks pituitary GnRH receptors [113]. It is effective for advanced (hormone-sensitive) prostate cancer [114]. In the phase-1 healthy-volunteer study of the drug, bradycardia was among the common adverse events; however, it did not exceed grade 2 [115]. Tamoxifen has been linked to bradyarrhythmias only rarely. The available evidence consists mainly of isolated case reports describing symptomatic sinus bradycardia in combination with marked QT prolongation, sometimes in the context of drug–drug interactions, with resolution after withdrawal of tamoxifen [116,117]. Other endocrine treatments used in oncology (such as those applied in endometrial or, rarely, ovarian cancer) show only limited evidence for an association with bradyarrhythmias. Because of the weak and inconsistent signal in the available literature, these therapies were not discussed in detail in this review.

### 3.13. CDK4/6 Inhibitors

This group of drugs acts by inhibiting cyclin-dependent kinases 4 and 6 (CDK4/6), disrupting the G1/S transition of the cell cycle [118]. These are most commonly used in HER-2-negative breast cancer therapy in combination with hormone therapy [119,120]. Within this drug class, the most consistent ECG safety signal is QT-interval prolongation [9].

Reports of clinically significant bradyarrhythmias include a case series describing two women on ribociclib or abemaciclib who developed new-onset, second-degree type II atrioventricular block requiring permanent pacemaker implantation [121]. A prospective Holter ECG study of 42 patients receiving ribociclib found no significant changes in rhythm parameters over follow-up, suggesting a low early risk of clinically relevant arrhythmias [122].

### 3.14. EGFR Inhibitors

EGFR tyrosine kinase inhibitors (EGFR-TKIs) inhibit the intracellular kinase activity of the epidermal growth factor receptor (EGFR), thereby downregulating downstream signaling pathways (rat sarcoma viral oncogene homolog (RAS)–rapidly accelerated fibrosarcoma (RAF)–mitogen-activated extracellular signal-regulated kinase (MEK)–extracellular signal-regulated kinase (ERK) and phosphoinositide 3-kinase (PI3K)–protein kinase B (AKT)–mechanistic target of rapamycin (mTOR) pathways) that control cell proliferation and survival in EGFR-dependent tumors [123]. Gefitinib and erlotinib are first-generation, reversible EGFR-TKIs, whereas osimertinib is a third-generation irreversible EGFR-TKI [124,125]; the primary therapeutic use of all three agents is in non-small-cell lung cancer (NSCLC) with activating EGFR mutations [123,126]. Across the class, QT-interval prolongation is the most consistently reported electrophysiologic cardiotoxicity; ischemic events have been described with earlier-generation TKIs (often in specific contexts or combinations), but a distinct bradyarrhythmic signal is also perceptible in the literature [127]. Regarding bradyarrhythmias, evidence for osimertinib and erlotinib consists mainly of isolated case reports or small series [128,129], whereas in a gefitinib cohort, sinus bradycardia occurred in 6.6% of patients [130].

### 3.15. Plant Alkaloids

Plant alkaloids constitute a broad class of cytotoxic agents used across multiple malignancies.

Here, we focus on selected representatives—irinotecan (camptothecin; topoisomerase I inhibitor), vincristine (vinca alkaloid), and the taxanes (paclitaxel)—to summarize bradyarrhythmia signals relevant to cardio-oncology.

Irinotecan is a topoisomerase I inhibitor [131]. Its primary use is in the treatment of colorectal cancer (colon and rectum) [132], and less commonly in gastric [133], esophageal [134], pancreatic [135], and lung cancers [136], as well as pediatric CNS (central nervous system) tumors [137,138]. In the literature, only isolated case reports describe the occurrence of bradycardia following irinotecan administration [139,140]. In phase II trials, bradycardia was an infrequent manifestation, observed in approximately 5% of patients who experienced irinotecan-induced cholinergic syndrome [141]. The leading theory explaining irinotecan-induced bradycardia used to be cholinergic effect through inhibition of acetylocholinesterase [142], although some animal studies appear to contradict this [143]. The same authors indicate that irinotecan promotes a parasympathetic discharge to peripheral organs, mediated by capsaicin-sensitive vagal afferent fibers, and that serotonin 5-HT3 (5-hydroxytryptamine type 3) receptors are implicated in the genesis of the vago-vagal reflex triggered by irinotecan [144]. Because of the multiple cholinergic adverse effects associated with irinotecan, including bradycardia, prophylactic or therapeutic administration of 0.25–1 mg of atropine intravenously or subcutaneously during treatment should be considered [139].

Vincristine is an antimitotic agent from the class of vinca alkaloids. Its action involves a direct effect on microtubules, leading to the loss or abnormal formation of the mitotic spindle and arresting cell division at metaphase [145]. It is used primarily in the treatment of acute lymphoblastic leukemia (ALL) [146], Hodgkin lymphoma [147], Wilms tumor [148], and rhabdomyosarcoma [149]. In the literature, case reports of bradycardia have been described [150], and some authors attribute the mechanism to vinca-alkaloid-induced neuropathy involving the autonomic nervous system [150]. At present, there is no effective prophylaxis for this neuropathy [151].

Paclitaxel is a taxane originally isolated from the bark of the Pacific yew [152]. Yew poisoning has been associated with cases of ventricular tachycardia and sudden cardiac death [152]. Paclitaxel’s mechanism of action involves binding to tubulin and stabilizing microtubules, which leads to the formation of a rigid, abnormal mitotic spindle and arrests cell division at the metaphase/anaphase transition [153]. It is used mainly in the treatment of ovarian, breast, and lung cancers, as well as Kaposi sarcoma [153]. A frequent rhythm disturbance associated with this drug is transient, asymptomatic bradycardia—in one study of 45 patients, 29% experienced asymptomatic bradycardia [154], whereas regulatory labels from the FDA report paclitaxel-associated bradycardia in ~3% of patients during the first 3 h of infusion (about 1% of treatment courses) [155]. Episodes of atrioventricular block, both transient and persistent, have also been reported with paclitaxel [154,156].

### 3.16. Platinum Compounds

Platinum-based cytotoxics are foundational in solid-tumor therapy; beyond ischemic risk, they have been linked to occasional bradyarrhythmias, often in the context of electrolyte disturbances. Here, we focus on cisplatin as the prototypical agent to outline the clinical pattern, and practical management.

Cisplatin is an inorganic chemotherapeutic agent. Its mechanism of action involves forming cross-links between adjacent DNA strands or within the same strand, which in turn prevents DNA replication and cell division [157]. Cardiotoxicity of cisplatin is most commonly associated with a significant risk of developing coronary artery disease [9].

Case reports describe cisplatin-induced bradycardia that recurs during subsequent administrations of the drug [158,159,160]. Episodes of complete atrioventricular block during infusion have also been reported [161]. The arrhythmias associated with cisplatin are most commonly attributed to disturbances in magnesium homeostasis [162]. A direct effect on the cardiac conduction system, including the sinoatrial node, has also been hypothesized [162], supported by the proven inotropic effect of cisplatin [163]. In case of bradyarrhythmia occurrence, electrolyte disturbances, primarily hypomagnesemia [164], should be promptly assessed and corrected, as they are a common and reversible trigger of bradyarrhythmia.

### 3.17. Alkylating Agents

Alkylating agents are therapeutic mainstays in oncology; the risk of bradyarrhythmia is low but documented. This section focuses on cyclophosphamide, outlining the clinical phenotype, its dose dependence (predominantly with high-dose regimens), and implications for management.

Cyclophosphamide is an alkylating agent. It is used mainly in the treatment of non-Hodgkin lymphomas [165], breast cancer [166], and multiple myeloma [167]. According to the summary of product characteristics, the most frequently reported cardiotoxic adverse events are atrial fibrillation (frequent, 1–10%) and heart failure (rare, <1%) [9].

When it comes to bradyarrhythmias, cases of complete heart block have been reported during infusion or within a few hours after cyclophosphamide administration, and the risk appears to be dose-related, with all reported cases occurring after high-dose therapy [168,169,170]. In a study of 39 women with breast cancer receiving high-dose cyclophosphamide, four experienced transient episodes of second-degree and high-grade atrioventricular block [171]. The rhythm disturbances occurred within hours up to a few days after drug administration. They did not recur on post-treatment Holter ECG monitoring. The authors suggested that severe vomiting may have contributed to the conduction abnormalities and recommended prophylaxis against emesis [171]. Sinus bradycardia is commonly listed as a potential adverse effect; however, robust data on its incidence is lacking [172].

### 3.18. mTOR Inhibitors

Bradyarrhythmias are rarely reported with mTOR inhibitors, largely confined to isolated case reports. This section focuses on everolimus as a representative agent to frame the subsequent discussion of bradyarrhythmias in this therapeutic class.

Everolimus is an mTORC1 (mechanistic target of rapamycin complex 1) inhibitor that suppresses cell proliferation and angiogenesis and can promote apoptosis in cancer cells [173]. Beyond its broad use in transplantation, everolimus has demonstrated antitumor efficacy in pancreatic neuroendocrine tumors [174], hormone receptor-positive/HER2-negative breast cancer [175], and renal cell carcinoma after failure of VEGFR-targeted tyrosine-kinase inhibitors [176]. The most commonly observed cardiovascular-relevant toxicities with everolimus are metabolic—hyperglycemia and dyslipidemia—along with hypertension [177]. Bradyarrhythmias with mTOR inhibitors appear to be rare; only isolated cases have been reported, often in the context of combination therapy or in transplant recipients, making causality uncertain [178].

### 3.19. Corticosteroids

Glucocorticoids are used in a wide range of clinical conditions and also occupy an important role in oncology, where they are administered both as part of antineoplastic treatment in selected hematologic malignancies and as supportive therapy, including for the prevention of chemotherapy-induced nausea and vomiting, hypersensitivity reactions, cytokine release syndromes, and tumor- or treatment-related complications such as cerebral edema [179,180]. Available evidence indicates, however, that high-dose corticosteroids may also induce cardiac rhythm and conduction disturbances. In a prospective study of 52 patients with multiple sclerosis receiving intravenous methylprednisolone at a dose of 1000 mg, Vasheghani-Farahani et al. demonstrated that pulse therapy may lead to a broad spectrum of arrhythmias: although sinus tachycardia was the most frequent finding, sinus bradycardia was also observed in 41.9% of patients, and sinoatrial conduction disturbances, including sinus arrest and sinoatrial block, were additionally reported (*n* = 16; 9.6% occurring exclusively after steroid administration), suggesting that high-dose steroids may substantially affect this level of cardiac conduction [181]. In a controlled study including 30 patients with pemphigus who were receiving corticosteroid pulse therapy for the first time, Jain et al. likewise observed bradycardia in approximately one third of patients, further supporting the concept that steroid-related bradyarrhythmia represents a reproducible pharmacologic phenomenon rather than an observation limited to a single disease [182]. Particularly relevant data in the oncologic setting were provided by Duffy et al., who, in a cohort of 153 pediatric patients, showed that during induction chemotherapy for acute lymphoblastic leukemia and lymphoblastic lymphoma, exposure to corticosteroids (prednisone 30 mg/m^2^/dose twice daily or dexamethasone 3–5 mg/m^2^/dose twice daily) was associated with a reduction in heart rate in 150 patients (98%). Heart rate of first percentile for age and lower developed in 90 (59%) patients. The lowest heart rate was observed, on average, after seven administered doses. No clinical complications related to bradycardia were identified, and most cases resolved during follow-up, suggesting that in pediatric hemato-oncology, steroid-associated bradycardia is common but usually transient and clinically benign [183]. These findings indicate that glucocorticoids should be considered a potential contributing factor to bradyarrhythmias in oncology patients, most commonly in the form of transient sinus bradycardia and, in some cases, possibly sino-atrial conduction disturbances.

### 3.20. Radiotherapy (RT)

Radiotherapy remains one of the fundamental modalities of modern cancer treatment and is widely used across a broad spectrum of malignancies. Hodgkin lymphoma, breast cancer, and lung cancer are among the common malignancies that often require radiotherapy, which may result in incidental cardiac irradiation. Consequently, cardiovascular toxicity resulting from cardiac exposure during thoracic irradiation constitutes a clinically relevant problem that often manifests years or even decades after treatment completion.

Radiation-induced cardiac injury comprises a broad spectrum of cardiovascular pathologies, whose clinical presentation depends on the time from exposure, the cardiac structures irradiated, the delivered dose, and pre-existing cardiovascular risk factors. Early manifestations are generally uncommon and are predominantly inflammatory, including acute pericarditis, while acute myocarditis or myopericarditis appears to be rare and are mainly described in case reports [184,185,186]. In contrast, late radiation-associated cardiac disease is clinically more relevant and encompasses coronary artery disease, heart failure, valvular heart disease, pericardial disease, arrhythmias, and conduction system abnormalities [187,188].

The mechanisms underlying radiation-induced conduction system disease have not been fully elucidated, but available evidence suggests a role for progressive fibrosis, microvascular injury, and structural remodeling of the myocardium and specialized conduction tissue. In an electrophysiological–pathological case report, Cohen et al. described a patient who developed clinically significant atrioventricular block 11 years after mantle radiotherapy for Hodgkin disease; the autopsy demonstrated marked arteriolosclerosis of the sinoatrial node and its approaches, as well as fibrosis involving the approaches to the atrioventricular node, the atrioventricular bundle, and both bundle branches [189]. Although direct imaging evidence linking septal fibrosis to post-radiotherapy bradyarrhythmias remains limited, cardiac magnetic resonance studies support the broader concept of radiation-induced myocardial fibrosis. In a contrast-enhanced MRI (magnetic resonance imaging) study of 24 patients treated with radiotherapy for esophageal cancer, Umezawa et al. found late gadolinium enhancement in 12 patients, with LGE (late gadolinium enhancement) present in 15.38% of myocardial segments exposed to approximately 40 Gy (gray) and 21.21% of segments exposed to approximately 60 Gy, but in none of the segments outside the radiation field, suggesting a dose-dependent pattern of radiation-associated myocardial injury [190].

Clinical evidence specifically linking radiotherapy to bradyarrhythmias is less robust than radiation-associated coronary artery disease, but several studies support the biological and clinical relevance of this phenomenon. In a cohort of 748 patients treated with radiotherapy for locally advanced non-small-cell lung cancer, grade ≥ 3 bradyarrhythmia occurred in 19 patients, and right coronary artery (RCA) irradiation, particularly when exceeding 25 Gy, was associated with subsequent bradyarrhythmia, suggesting that radiation dose to anatomically relevant cardiac substructures may interact with baseline cardiovascular vulnerability [191]. In long-term survivors of Hodgkin disease treated with chest radiotherapy, Adams et al. reported a high prevalence of subclinical conduction abnormalities; conduction defects were observed in 75% of patients, most commonly as an RSR′ pattern in the right precordial leads, while sinus bradycardia was present in three patients and one patient developed complete atrioventricular block [192]. Evidence from large breast cancer cohorts is more heterogeneous. In a French nationwide healthcare database sample of 3853 breast cancer patients, radiotherapy-treated patients had 28 permanent pacemaker implantations compared with 13 expected cases, corresponding to a standardized incidence ratio of 2.18, although the direct comparison with non-irradiated breast cancer patients was only borderline significant [193]. Conversely, in a Danish cohort of 44,423 women with early-stage breast cancer, 179 cardiac implantable electronic devices were implanted among 18,251 women who received radiotherapy and 401 among 26,172 women who did not receive radiotherapy; after adjustment, radiotherapy was not associated with an increased risk of device implantation, with an incidence rate ratio of 1.13 (95% CI confidence interval 0.93–1.38) [194]. The negative Danish cohort should be interpreted with caution, as patients not receiving radiotherapy were older and may therefore have had a higher background risk of degenerative conduction-system disease and device implantation. Taken together, these data suggest that clinically overt radiation-associated bradyarrhythmias are uncommon and likely represent late events, but they may become relevant in selected patients, particularly when thoracic irradiation involves conduction-related cardiac substructures or occurs in individuals with pre-existing cardiovascular disease.

Prevention of radiation-associated bradyarrhythmias is based primarily on careful radiotherapy planning and minimization of unnecessary cardiac exposure, rather than on a specific pharmacological strategy. In this context, dosimetric studies have shown that the sinoatrial and atrioventricular nodes may receive incidental radiation during thoracic or mediastinal radiotherapy and that treatment technique can influence the dose delivered to these conduction system structures [195]. However, clinically validated methods for selecting patients who would benefit from dedicated conduction-system-sparing approaches, as well as specific dose constraints for the sinoatrial and atrioventricular nodes, remain to be established.

### 3.21. Combination Therapies

Bradyarrhythmias have been reported in several oncologic combination regimens; however, the available evidence is heterogeneous and usually does not allow for a clear distinction between a true combination effect and toxicity attributable to a single component of the regimen. A bradyarrhythmic signal has been observed, among others, in thalidomide-based regimens, particularly when thalidomide is combined with dexamethasone. In a phase III trial in newly diagnosed multiple myeloma, thalidomide 200 mg/day combined with dexamethasone was associated with a higher overall rate of grade ≥ 3 toxicity compared with dexamethasone alone; however, grade ≥ 3 sinus bradycardia was uncommon and was not emphasized as a frequent adverse event [196]. In contrast, in AL amyloidosis, thalidomide plus dexamethasone, with thalidomide escalated up to 400 mg/day, was associated with symptomatic bradycardia in 26% of patients [197]. This discrepancy suggests that the bradycardic signal may be highly dependent on both thalidomide exposure and disease context, with the higher rate observed in AL amyloidosis potentially reflecting not only greater drug exposure but also disease-specific susceptibility, including possible cardiac and autonomic involvement.

Isolated severe events have also been reported in other combination regimens. In a phase Ib dose-finding study of intravenous panobinostat combined with docetaxel and prednisone in hormone-refractory prostate cancer, a single case of grade 4 bradycardia was reported as a dose-limiting toxicity; however, this event was isolated and was not highlighted as a principal toxicity of the regimen [198]. In hormone receptor-positive, HER2-negative breast cancer, giredestrant administered alone or in combination with palbociclib and/or a luteinizing hormone-releasing hormone agonist was associated with dose-dependent asymptomatic bradycardia, but without clinically significant changes in cardiac-related outcomes, supporting a generally mild and manageable heart-rate effect in this setting [199].

Cited data indicate that bradyarrhythmias in oncologic combination regimens are plausible and may occasionally be severe; however, the current evidence remains largely signal-generating. The most reproducible signal appears to involve thalidomide-based regimens, whereas observations related to others mentioned above are more limited and do not currently allow for the identification of a consistent, high-risk combination pattern for clinically significant bradyarrhythmias.

## 4. Monitoring

Ambulatory ECG monitoring may play an important role in detecting rhythm and conduction disturbances, including bradyarrhythmias, in selected oncology patients. However, the available evidence does not currently support routine prolonged ECG monitoring in all patients starting anticancer therapy. The strongest bradycardic signal is observed with ALK inhibitors and immunomodulatory drugs, for which the 2022 ESC cardio-oncology guidelines recognize sinus bradycardia as a treatment-related toxicity and recommend Holter ECG monitoring in symptomatic patients to exclude significant sinus pauses [9]. In most of ALK inhibitors, Alecitinib for example, bradycardia may develop relatively early but not necessarily at baseline: in a real-world cardio-oncology cohort, the median time to bradycardia was three weeks, and 91% of cases occurred within the first three months of treatment [28]. Therefore, a single resting ECG, and even one-time 24 h Holter monitoring, may be insufficient to assess intermittent or delayed rhythm and conduction abnormalities.

Longer ambulatory monitoring modalities, such as 7–14-day ECG patches, may increase diagnostic yield compared with conventional 24 h Holter monitoring, particularly when paroxysmal events are suspected. In non-oncologic populations referred for arrhythmia evaluation, 14-day patch monitoring detected more arrhythmic events than 24 h Holter monitoring and significantly improved the detection of paroxysmal arrhythmias, including clinically relevant pauses [200,201]. Accordingly, prolonged ambulatory ECG monitoring should be considered in patients starting therapies with documented bradycardic potential, particularly in the presence of baseline ECG abnormalities, symptoms suggestive of bradyarrhythmia, pre-existing conduction disease, or concomitant use of rate-lowering drugs. Wearable and smartwatch-based ECG technologies may complement conventional ambulatory ECG monitoring, particularly when tachycardia-bradycardia syndrome with atrial fibrillation is suspected [202]. At present, this approach should be considered as risk-adapted monitoring rather than a universal screening strategy for all oncology patients.

## 5. Discussion

Bradyarrhythmias emerging during contemporary cancer therapy represent a heterogeneous spectrum, ranging from transient asymptomatic sinus bradycardia to high-grade atrioventricular block requiring temporary or permanent pacing. The strength of evidence, however, varies markedly across drug classes: for only a limited subset of agents (e.g., ALK inhibitors, thalidomide, pazopanib, paclitaxel, and 5-fluorouracil) incidence can be quantified in prospective studies or pooled trial datasets, whereas for many newer targeted therapies and cytotoxics the signal largely derives from pharmacovigilance reports and case-based literature. Importantly, the bradyarrhythmic phenotype appears class specific. ALK-TKIs commonly induce dose-dependent sinus bradycardia, typically reversible after treatment interruption or dose reduction and seldom progressing to advanced conduction disease, pointing toward a predominantly functional sinoatrial mechanism (including If inhibition demonstrated for crizotinib) [25,203]. In contrast, immune checkpoint inhibitors represent the highest-acuity setting, in which bradyarrhythmias often accompany ICI-myocarditis, and high-grade AV block is both frequent and prognostically adverse [13,204].

From a clinical perspective, these class-dependent patterns argue for proactive, risk-adapted monitoring. Baseline ECG assessment, careful review of concomitant bradycardic medications, and electrolyte surveillance are particularly important before and during therapies with known nodal or conduction effects. Management should follow ESC cardio-oncology and pacing guidance, using drug-specific algorithms where available (e.g., for ALK-TKIs) and considering early temporary pacing in unstable patients or those with high-grade AV block. A key limitation of the current evidence base is underreporting rhythm phenotypes in pivotal oncology trials, often without systematic ambulatory monitoring. Prospective cardio-oncology studies incorporating continuous rhythm surveillance could clarify true incidence, predictors, and long-term outcomes, thereby facilitating safer continuation of effective anticancer therapy.

## 6. Conclusions

Bradyarrhythmias associated with oncologic treatment are clinically meaningful but unevenly characterized across anticancer drug classes. They range from transient, asymptomatic sinus bradycardia to high-grade atrioventricular block requiring temporary or permanent pacing. The most severe bradyarrhythmic phenotype appears to occur in the setting of immune checkpoint inhibitor-associated myocarditis, where high-grade or complete atrioventricular block may accompany a fulminant and potentially life-threatening inflammatory cardiotoxicity.

More reproducible signals for sinus bradycardia were identified with ALK inhibitors, particularly crizotinib and alectinib, as well as with thalidomide, antimetabolites—especially 5-fluorouracil-based regimens—taxanes, and high-dose corticosteroids. Bradyarrhythmias were also reported with proteasome inhibitors, BTK inhibitors, anthracyclines, platinum compounds, high-dose cyclophosphamide, endocrine therapies, EGFR inhibitors, RAF/MEK inhibitors, VEGFR-TKIs, and mTOR inhibitors; however, the strength of evidence varied substantially, ranging from regulatory or cohort-based data to isolated case reports.

From a clinical perspective, recognition of class-specific patterns is essential. Baseline ECG assessment, rhythm monitoring in symptomatic or higher-risk patients; electrolyte surveillance and timely identification of potential drug interactions may help minimize unnecessary interruption of effective cancer therapy. Temporary or permanent pacing should be reserved for clinically significant or persistent high-grade conduction disease according to standard pacing guidance.

Combination regimens add further complexity. Available reports suggest that clinically significant bradyarrhythmias, including rare grade 4 events, may occur during selected oncologic combination therapies. However, the current evidence usually does not allow for a clear distinction between toxicity attributable to a single component and a true additive or synergistic bradyarrhythmic effect. Therefore, cumulative bradyarrhythmic risk from combining agents with bradycardic potential remains biologically plausible but should be regarded as signal-generating and requiring prospective validation.

## Figures and Tables

**Table 1:** Grading of bradyarrhythmic events as stated in “Common Terminology Criteria for Adverse Events (CTCAE) v6.0 (MedDRA 28.0)”, published 22 July 2025 by the Cancer Therapy Evaluation Program Division of Cancer Therapy and Diagnosis National Cancer Institute National Institutes of Health U.S. Department of Health and Human Services.

Bradyarrhythmia Type	Definition	Grade 1	Grade 2	Grade 3	Grade 4
Sinus bradycardia	A disorder characterized by a dysrhythmia with a heart rate less than 60 beats per minute that originates in the sinus node.	Asymptomatic, intervention not indicated	Symptomatic, intervention not indicated; change in medication initiated	Symptomatic, intervention indicated	Life-threatening consequences; urgent intervention indicated
Mobitz II grade AV block (type 1 and 2)	A disorder characterized by a dysrhythmia with relatively constant PR interval prior to the block of an atrial impulse. This is the result of intermittent failure of atrial electrical impulse conduction through the atrioventricular (AV) node to the ventricles.	Asymptomatic, intervention not indicated	Symptomatic; medical intervention indicated	Symptomatic and incompletely controlled medically, or require a device (e.g., pacemaker)	Life-threatening consequences; urgent intervention indicated
Complete AV block	A disorder characterized by a dysrhythmia with complete failure of atrial electrical impulse conduction through the AV node to the ventricles.	-	Non-urgent intervention indicated	Symptomatic and incompletely controlled medically, or controlled with device (e.g., pacemaker); new onset	Life-threatening consequences; urgent intervention indicated

**Table 2:** The table summarizes incidence, type and management of studied drug classes. Abbreviations/Frequency categories: c, case reports only or frequency not estimable (no reliable percentage available; possibly <0.1%); +, rare/uncommon (≥0.1% to <1%); ++, common (≥1% to <10%); +++, very common (≥10%). * higher incidence when myocarditis occurs; ** high-grade AV block is mainly case-based, but a high-dose chemotherapy cohort reported transient second-degree/high-grade AV block in 4/39 patients; *** 3% in FDA label; 29% in small cohort study; **** 2% to 12% in medium-sized studies.

Drug	Reported Frequency	Bradyarrhythmia Type	Specific Management	Strength of Evidence
Immune checkpoint inhibitors				
Pembrolizumab	c/+++ *	Complete AV block	Intensive treatment of myocarditis if present-	Low-to-moderate; mainly case reports/case-based reviews and retrospective ICI-myocarditis cohorts, with strongest support for complete AV block occurring in ICI-myocarditis.
Anaplastic lymphoma kinase inhibitors				
Crizotinib	+++	Sinus bradycardia	Specific dose reduction protocol	High; FDA label + large clinical-trial safety dataset
Alectinib	+++	Sinus bradycardia	Specific dose reduction protocol	High; FDA label + large clinical-trial safety dataset
Ceritinib	++	Sinus bradycardia	Specific dose reduction protocol	Moderate; FDA label + pooled clinical-trial safety data, but low event frequency
Proteasome inhibitors				
Bortezomib	c	Sinus node dysfunction Atrioventricular block	-	-
Immunomodulatory Imide Drugs				
Thalidomide	++	Sinus bradycardia Complete atrioventricular block (cases)	-	-
Lenalidomide	c	Sinus bradycardia	-	-
Pomalidomide	c	Sinus bradycardia	-	-
VEGF inhibitors (VEGFR-TKI)				
Pazopanib	++	Sinus bradycardia	-	-
RAF/MEK inhibitors				
Trametinib	++	Sinus bradycardia	-	-
HER2-targeted therapies				
Trastuzumab	c	Sinus bradycardia	-	-
Bruton tyrosine kinase inhibitors (BTK-i)				
Ibrutinib	c	Sinus node dysfunction High grade AV block	-	-
Anthracyclines				
Doxorubicin	++	Sinus bradycardia Complete atrioventricular block (cases)	-	-
BCR-ABL TKI				
Ponatinib	+	Sinus bradycardia	-	-
Dasatinib	c	Complete AV block	-	-
Nilotinib	c	Sinus bradycardia	-	-
Endocrine therapies				
Triptorelin	c	Complete AV block	-	-
Relugolix	+	Sinus bradycardia (up to grade 2)	-	-
Tamoxifen	c	Sinus bradycardia	-	-
Alkylating agents				
Cyclophosphamide	c/+ **	2nd degree, 3rd degree and high-grade atrioventricular block	-	-
CDK4/6 inhibitors				
Ribociclib	c	Atrioventricular block 2nd degree	-	-
Abemaciclib	c	Atrioventricular block 2nd degree	-	-
EGFR inhibitors				
Gefitinib	++	Sinus bradycardia	-	-
Erlotinib	c	Sinus bradycardia	-	-
Osimertinib	c	Sinus bradycardia	-	-
Plant alkaloids				
Irinotecan	++	Sinus bradycardia	Atropin 0.25–1 mg i.v. for acute cholinergic symptomes; prophylaxis may be considered after prior cholinergic symptoms	Low-to-moderate; label/formulary-supported for cholinergic syndrome, but bradycardia evidence mainly case reports and infrequent trial signal
Vincristine	c	Sinus bradycardia	-	-
Paclitaxel	++/+++ ***	Sinus bradycardia Complete AV block (cases)	-	-
Platinum compounds				
Cisplatin	c	Sinus bradycardia	Management of magnesium serum concentration	Low; case-report evidence with mechanistic support for magnesium-related contribution.
Antimetabolites				
5-fluorouracil	++/+++ ****	Sinus bradycardia	-	-
Cytarabine	++	Sinus bradycardia	-	-
mTOR inhibitors				
Everolimus	c	Sinus bradycardia	-	-
Glucocortycosteroids				
High-dose/pulse treatment	+++	Sinus bradycardia Sino-atrial conduction disturbances	-	-

## Data Availability

No new data was created for this work.

## Abbreviations

5-FU	5-fluorouracil
5-HT3	5-hydroxytryptamine type 3
ADT	androgen deprivation therapy
AKT	protein kinase B
ALK	anaplastic lymphoma kinase
ALK-TKI/ALK-TKIs	anaplastic lymphoma kinase tyrosine kinase inhibitor(s)
ALL	acute lymphoblastic leukemia
ATP	adenosine triphosphate
AV	atrioventricular
BCR-ABL/BCR-ABL1	breakpoint cluster region–Abelson/BCR-ABL1 oncoprotein
BRAF	B-Raf proto-oncogene serine/threonine kinase
BTK	Bruton tyrosine kinase
BTK-i/BTKi	Bruton tyrosine kinase inhibitor(s)
CDK4/6	cyclin-dependent kinases 4 and 6
CI	confidence interval
CML	chronic myeloid leukemia
CNS	central nervous system
CTCAE	Common Terminology Criteria for Adverse Events
CTLA-4	cytotoxic T-lymphocyte-associated protein 4
EGFR	epidermal growth factor receptor
EGFR-TKI/EGFR-TKIs	epidermal growth factor receptor tyrosine kinase inhibitor(s)
ERK	extracellular signal-regulated kinase
ESC	European Society of Cardiology
FAERS	FDA Adverse Event Reporting System
FDA	U.S. Food and Drug Administration
GnRH	gonadotropin-releasing hormone
Gy	gray
HER2/HER-2	human epidermal growth factor receptor 2
HGFR	hepatocyte growth factor receptor
ICI/ICIs	immune checkpoint inhibitor(s)
If	funny current
IgG1	immunoglobulin G1
IgG4	immunoglobulin G4
IMiDs	immunomodulatory imide drugs,
IMT	inflammatory myofibroblastic tumor
LGE	late gadolinium enhancement
LH	luteinizing hormone
LH/FSH	luteinizing hormone/follicle-stimulating hormone
LV	left ventricular
LVEF	left ventricular ejection fraction
MACE	major adverse cardiovascular events,
MAPK	mitogen-activated protein kinase
MedDRA	Medical Dictionary for Regulatory Activities
MEK	mitogen-activated extracellular signal-regulated kinase
MET	MET proto-oncogene/MET receptor tyrosine kinase
MET/HGFR	MET/hepatocyte growth factor receptor
MRI	magnetic resonance imaging
mTOR	mechanistic target of rapamycin
mTORC1	mechanistic target of rapamycin complex 1
NK	natural killer
NSCLC	non-small-cell lung cancer
OPTIC	Optimizing Ponatinib Treatment in CML
PACE	Ponatinib Ph+ ALL and CML Evaluation
PD-1	programmed cell death protein 1
PD-L1	programmed death-ligand 1
Ph+/Ph-positive	Philadelphia chromosome-positive
PI3K	phosphoinositide 3-kinase
QT	QT interval
QTc	corrected QT interval
RAF	rapidly accelerated fibrosarcoma
RAS	rat sarcoma viral oncogene homolog
RCC	renal-cell carcinoma
RCA	right coronary artery
ROS1	ROS proto-oncogene 1 receptor tyrosine kinase
RT	radiotherapy,
STS	soft-tissue sarcoma
TKI/TKIs	tyrosine kinase inhibitor(s)
TNF-α/TNF-alpha	tumor necrosis factor alpha
VEGF	vascular endothelial growth factor
VEGFR	vascular endothelial growth factor receptor
VEGFR-TKI/VEGFR-TKIs	vascular endothelial growth factor receptor tyrosine kinase inhibitor(s)
